# Normal red blood cells’ shape stabilized by membrane’s in-plane ordering

**DOI:** 10.1038/s41598-019-56128-0

**Published:** 2019-12-24

**Authors:** L. Mesarec, W. Góźdź, A. Iglič, V. Kralj-Iglič, E. G. Virga, S. Kralj

**Affiliations:** 1 Laboratory of Biophysics, Faculty of Electrical Engineering, University of Ljubljana, 1000 Ljubljana, Slovenia; 2 Institute of Physical Chemistry, Polish Academy of Sciences, 01-224 Warsaw, Poland; 3 Laboratory of Mass Spectrometry and Proteomics, Institute of Biosciences and BioResources, National Research Council of Italy, Napoli, 80132 Italy; 4 Laboratory of Clinical Biophysics, Faculty of Health Sciences, University of Ljubljana, 1000 Ljubljana, Slovenia; 5 Laboratory of Clinical Biophysics, Faculty of Medicine, University of Ljubljana, 1000 Ljubljana, Slovenia; 6 Department of Mathematics, University of Pavia, Via Ferrata 5, 27100 Pavia, Italy; 7 Department of Physics, Faculty of Natural Sciences and Mathematics, University of Maribor, 2000 Maribor, Slovenia; 8 Condensed Matter Physics Department, Jožef Stefan Institute, 1000 Ljubljana, Slovenia

**Keywords:** Biological physics, Topological defects

## Abstract

Red blood cells (RBCs) are present in almost all vertebrates and their main function is to transport oxygen to the body tissues. RBCs’ shape plays a significant role in their functionality. In almost all mammals in normal conditions, RBCs adopt a disk-like (discocyte) shape, which optimizes their flow properties in vessels and capillaries. Experimentally measured values of the reduced volume (*v*) of stable discocyte shapes range in a relatively broad window between *v* ~ 0.58 and 0.8. However, these observations are not supported by existing theoretical membrane-shape models, which predict that discocytic RBC shape is stable only in a very narrow interval of *v* values, ranging between *v* ~ 0.59 and 0.65. In this study, we demonstrate that this interval is broadened if a membrane’s in-plane ordering is taken into account. We model RBC structures by using a hybrid Helfrich-Landau mesoscopic approach. We show that an extrinsic (deviatoric) curvature free energy term stabilizes the RBC discocyte shapes. In particular, we show on symmetry grounds that the role of extrinsic curvature is anomalously increased just below the nematic in-plane order-disorder phase transition temperature.

## Introduction

**Figure 1:**
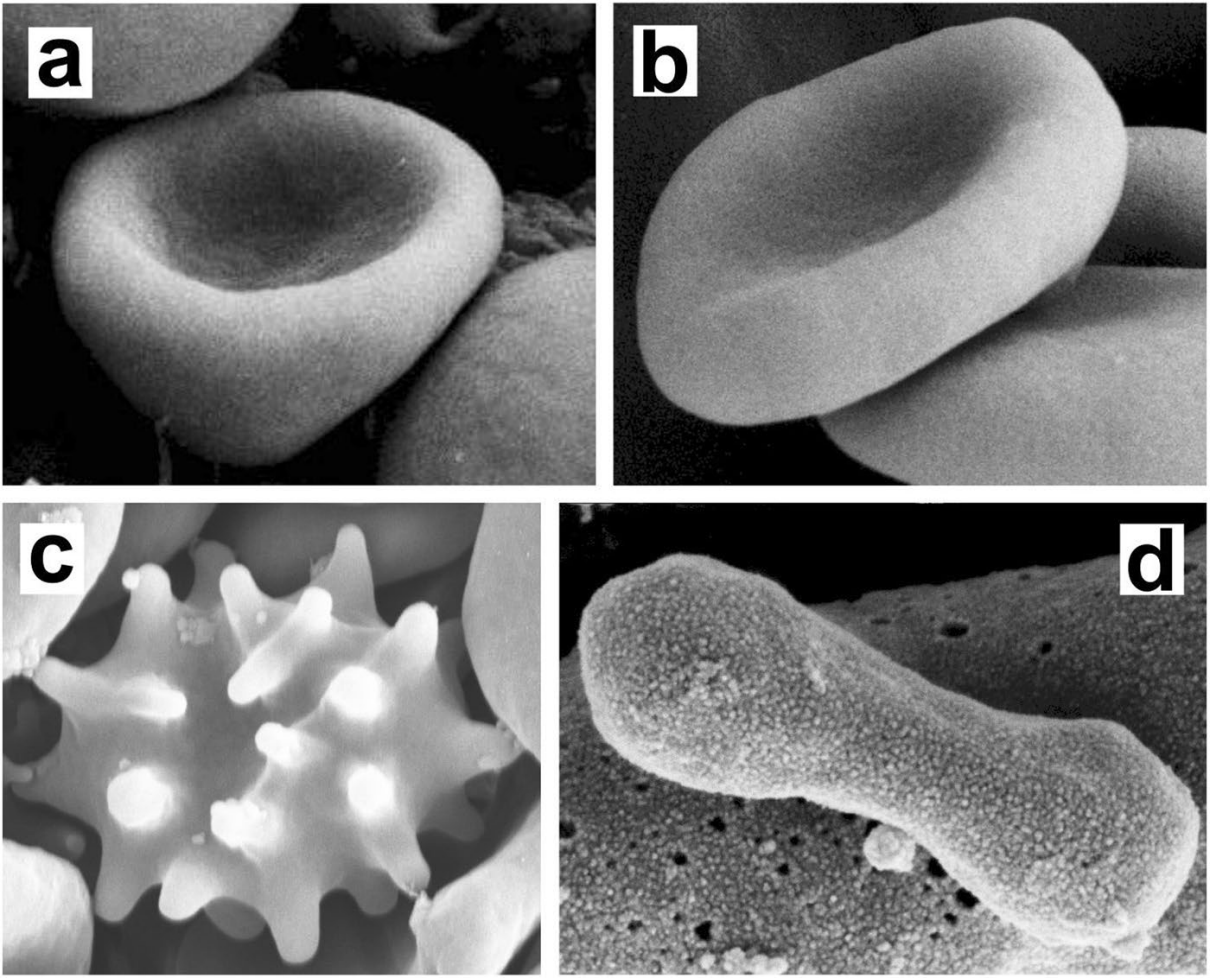
Scanning electron microscope images of (**a)** open stomatocyte, (**b)** discocyte, (**c)** echinocyte, and (**d)** prolate membrane shape. Structures a, b and c show RBCs. Note that prolate shapes (**d**) are typically observed in extracellular vesicles.

Red blood cells (RBCs) are biological cells playing a vital role in all vertebrates. In mammals, their main role is to transport oxygen to all parts of a body’s tissue. The normal shape of RBCs is a biconcave discoid (Fig. 1b) which can be transformed in other shapes, such as cup-shaped stomatocyte (Fig. 1a) or spiculated echinocyte (Fig. 1c)^1–8^. The discocyte RBC shape is invaginated in the center and torus-like at the rim. The meridian cross section has a dumb-belled shape. Optimal RBCs flow and their carrying and transport capabilities in “healthy” conditions coincide with discocyte RBC shape^9^, while in pathological conditions or in patients using drugs, a larger number of RBCs may have also stomatocyte or echinocyte shapes.

The key geometric parameter controlling the stability of discocyte RBC shapes is the reduced volume *v* = *V*/*V*_0_. Here *V* stands for the RBC volume and ${V}_{0}=4\pi {R}^{3}/3$ represents the volume of a spherical RBC with the same surface area, where $R=\sqrt{A/4\pi }$ is the radius of the sphere and *A* stands for the RBC surface area. In different mammals, the values of *v* in healthy cells possess a relatively broad range of values^1,5,10,11^. In humans, the reduced volumes of discocytes range within the interval $v\in [{v}_{1} \sim 0.58,{v}_{2} \sim 0.81]$ ^1^. However, the actual broad range of *v* for which stable disk-like RBC shapes exist cannot be reproduced using the existing theoretical approaches^4,7,12–15^.

Recent investigations^16,17^ suggest that several key features of biological systems are dominated by geometry. Minimal models, which are capable of reproducing a rich variety of existing membrane structures, typically treat them as fluid structureless 2D curved thin films. A pioneering mesoscopic model was introduced by Helfrich^4,12^, where the variational parameter is the membrane curvature tensor $\underline{C}$, represented by its invariants, namely, the Gaussian curvature *K* and the mean curvature *H*. The Helfrich model predicts three qualitatively different RBC shapes upon varying of the reduced volume *v*: (i) stomatocytes (Fig. 1a), (ii) oblate discocytes (Fig. 1b) and (iii) prolate shapes (Fig. 1d). These shapes are stable in different regimes of *v* values: (i)*v* < *v*_1_, (ii) ${v}_{1}\le v\le {v}_{2}$, (iii) *v* > *v*_2_, where *v*_1_ ~ 0.59 and *v*_2_ ~ 0.65. Therefore, the window [*v*_1_, *v*_2_] of stable discocyte shapes is relatively narrow with respect to experimental observations. This window could be slightly widened by adding additional free energy contributions, bringing the mathematical model closer to realistic configurations, for instance, by taking into account the bi-layer structure of the membrane^15,18,19^. However, for sensible values of the relevant additional parameters, the observed stability range of *v* for discocyte shapes has so far not been reproduced. Note that prolate RBC shapes are not experimentally observed under normal conditions. Furthermore, echinocytic RBC shapes (Fig. 1c) could be reproduced if the shear deformation of the membrane skeleton and lateral redistribution of different membrane components would be included in the Helfrich model^6,20,21^. Because in this paper we do not consider the spiculated (echinocytic) RBC shapes, the shear elastic energy of the RBC membrane skeleton is for simplicity neglected in our theoretical approach.

**Figure 2:**
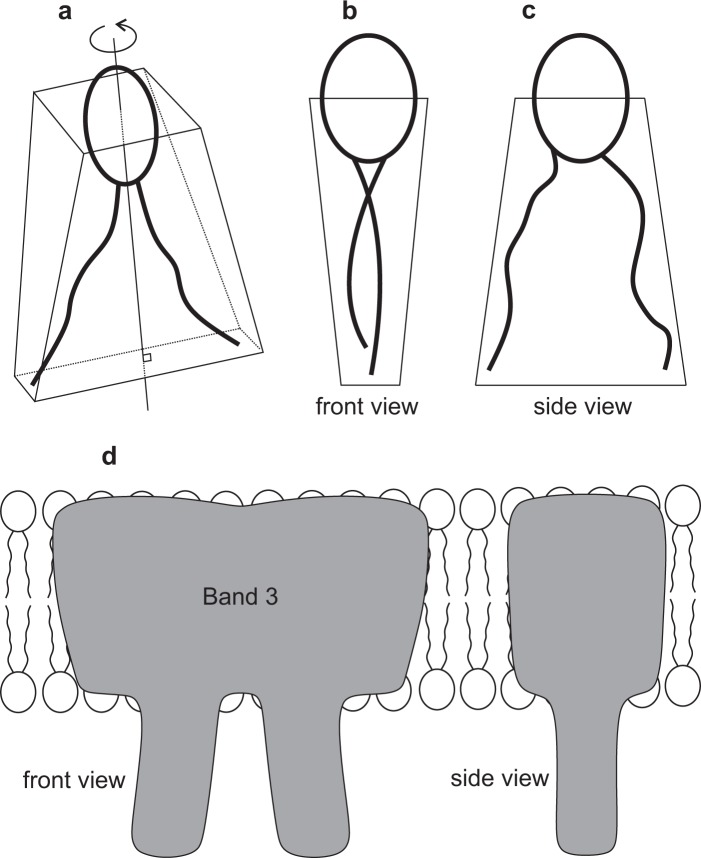
Possible origins of in-plane orientational ordering within membranes of red blood cells or extracellular vesicles. Effective nematic-type ordering could arise either from V-shaped stretched chains of phospholipids (top panel) or from anisotropic proteins like Band 3 proteins embedded within membranes.

In the following, we show that taking into account in-plane ordering of membranes could explain the experimentally observed broad stability window of *v* for discocyte shapes. Namely, biological membranes very likely possess in-plane ordering, especially in highly and anisotropically curved membrane regions^22–24^. Possible origins of nematic-type ordering in RBC membrane are presented in Fig. 2. For example, nematic ordering might be due to two flexible hydrocarbon chains of lipids^25–28^ or to anisotropic Band 3 proteins embedded within membranes^22,23,29–31^. Nematic order in biological membranes may also occur at high concentrations of membrane attached rod-like BAR domains, where the rotation of a single BAR domain becomes restricted due to direct/steric interaction with neighboring BAR domains^32^. Furthermore, the tails of lipid molecules in biological membranes may tilt relative to the surface normal and develop tilt and hexatic orientational ordering^33–35^. The in-plane orientational ordering in hexatic membranes with long range bond orientation order and short-range positional order has been also observed experimentally^36^. Low rotational diffusion of lipids may be driven by strong interactions at the lipid/water interface^37^. It has been also indicated within statistical-mechanical approach that in certain membrane regions, the average orientation of lipid molecules is not negligible in spite of the rotational movement of lipid molecules^25^.

In case of in-plane ordering, topological defects (TDs) unavoidably appear in non-toroidal topologies (see Supplementary Material). TDs are characterized by a discrete topological charge, which in 2D is equivalent to the winding number *m*^38,39^. Their total winding number in closed 2D geometries is determined by the Gauss-Bonnet and Poincare-Hopff theorems^40,41^. In case of nematic ordering, *m* can be multiple of half an integer. One commonly refers to TDs bearing positive and negative values of *m* as *defects* and *antidefects*, respectively.

Position and number of TDs are controlled by *intrinsic* and *extrinsic*^40,42,43^ curvature contributions to the elastic free energy. Most studies so far have expressed the elastic free energy penalties using covariant derivatives^44–49^. In such approaches, the extrinsic curvature contributions are discarded from the outset. For such cases, it has been demonstrated that regions exhibiting positive (negative) Gaussian curvature attract TDs bearing positive (negative) *m*. This is well embodied in the Effective Topological Charge Cancellation (ETCC) mechanism^50^. It applies to 2D geometries possessing surface patches exhibiting substantially different spatially averaged values of Gaussian curvature $\bar{K}$. To each such surface patch Δ*ζ*, characterised by $\bar{K}=-\,\frac{1}{\Delta \zeta }{\iint }_{\Delta \zeta }K{d}^{2}\overrightarrow{r}$, one assigns the effective topological charge $\Delta {m}_{eff}=\Delta m+\Delta {m}_{K}$. Here Δ*m* refers to the total charge of TDs, and $\Delta {m}_{K}=-\,\frac{1}{2\pi }{\iint }_{\Delta \zeta }K{d}^{2}\overrightarrow{r}$ is the so called smeared Gaussian curvature charge within Δ*ζ*. The ETCC mechanism claims that within each patch Δ*ζ*, there is a tendency to cancel Δ*m*_*eff*_. Note that the ETCC mechanism takes into account only the impact of the intrinsic curvature. However, Selinger *et al*.^42^ and Napoli and Vergori^43,51^ showed that in general, there is no justification to discard extrinsic-type terms. In studies addressing biological cells, such terms were considered already previously and referred to as deviatoric terms^22–26,52–54^. Minimal models^42^ suggest (see Supplementary Material) that intrinsic and extrinsic terms are weighted by elastic moduli of comparable strength. The extrinsic curvature is effective in points where the principal curvatures {*C*_1_, *C*_2_} are different and its strength increases with increased curvature deviator *D* = |*C*_1_ − *C*_2_|/2^24,52^. In several geometries, the impacts of intrinsic and extrinsic terms on positions of TDs might be antagonistic^42,51^.

In this contribution, we show that the extrinsic curvature, which becomes effective in structures exhibiting some kind of in-plane ordering, could anomalously increase the stability window of discocyte vesicle shapes. The in-plane order is assumed to appear spontaneously below the phase transition temperature *T*_*c*_. In particular, our model reveals that in general the extrinsic term dominates the orientational dependent free energy penalties just below *T*_*c*_, favoring discocyte shapes. Furthermore, we introduce curvature potentials which predict well the relative strength of the extrinsic curvature contributions, although indirectly, through the favourable position of topological defects.

## Results

In our study, we consider axisymmetric closed vesicles, which are treated as 2D elastic structureless sheets exhibiting nematic in-plane ordering. We henceforth refer to these objects as *nematic vesicles*. We model their configuration in terms of mesoscopic curvature and orientational ordering fields.

We use a 2D mesoscopic model in which the vesicle shape is determined by the curvature tensor $\underline{C}$ ^4,12^ and the orientational ordering is described by the 2D nematic tensor order parameter $\underline{Q}$ ^55,56^. In their eigenframes, these tensors are expressed as

1a $$\underline{C}={C}_{1}{\overrightarrow{e}}_{1}\otimes {\overrightarrow{e}}_{1}+{C}_{2}{\overrightarrow{e}}_{2}\otimes {\overrightarrow{e}}_{2},$$

1b $$\underline{Q}=\lambda (\overrightarrow{n}\otimes \overrightarrow{n}-{\overrightarrow{n}}_{\perp }\otimes {\overrightarrow{n}}_{\perp }).$$

Here the unit vectors {${\overrightarrow{e}}_{1},\,{\overrightarrow{e}}_{2}$} determine a local principal curvature frame at a surface point characterised by the surface normal $\overrightarrow{v}={\overrightarrow{e}}_{1}\times {\overrightarrow{e}}_{2}$, $\lambda \in [0,\,1/2]$ is the orientational order parameter, and $\overrightarrow{n}$ is the nematic director field indicating the direction of a local in-plane ordering, where states $\pm \,\overrightarrow{n}$ are physically equivalent and $|\overrightarrow{n}|=|{\overrightarrow{n}}_{\perp }|=1.$

We express the resulting free energy density per vesicle area as $f={f}_{H}+{f}_{c}+{f}_{e}$. Here *f*_*H*_ = $\frac{\kappa }{2}{(Tr\underline{C})}^{2}$ stands for the classical Helfrich vesicle curvature contribution^12^, which for a positive bending modulus *κ* resists to vesicle bending deformations. The nematic condensation contribution ${f}_{c}={\alpha }_{0}(T-{T}^{\ast })$ $Tr{\underline{Q}}^{2}+\frac{\beta }{4}$ ${(Tr{\underline{Q}}^{2})}^{2}$ enforces nematic orientational order below a critical temperature *T*_*c*_ that marks a second order phase transition. The quantities ${\alpha }_{0},\,\beta ,\,{T}^{\ast }$ are positive phenomenological constants. For a negligible vesicle curvature, the critical temperature is ${T}_{c}={T}^{\ast }$, below which the equilibrium degree of order is ${\lambda }_{0}=\sqrt{{\alpha }_{0}({T}^{\ast }-T)/\beta }.$ The elastic contribution consists of the intrinsic, ${f}_{int}=\frac{1}{2}{k}_{i}{|{\nabla }_{s}\underline{Q}|}^{2}$, and extrinsic, ${f}_{ext}={k}_{e}\underline{Q}\cdot {\underline{C}}^{2}$, contributions, where *k*_*i*_ and *k*_*e*_ stand for intrinsic and extrinsic elastic constants, which we set to be positive. ∇_*s*_ stands for the surface gradient operator^57^. Note, that the extrinsic term has similar impact as an external ordering field, which is present in regions where *C*_1_ ≠ *C*_2_. More modelling details are given in Supplementary Material.

We scale the tensor order parameter with respect to the bulk equilibrium order parameter and the curvature tensor and spatial coordinates with respect to *R*, i.e. $\underline{Q}\to \underline{Q}/{\lambda }_{0}$, $\underline{C}\to \underline{C}R$, ${\nabla }_{s}\to R{\nabla }_{s}$. The quantity $R=\sqrt{A/(4{\rm{\pi }})}$ describes the radius of a spherically shaped vesicle whose surface area *A* is the same as the surface area of the investigated vesicle. An additional length scale, playing an important role in our study is the nematic order parameter correlation length, which we express in the nematic phase as $\xi =\sqrt{{k}_{i}/({\alpha }_{0}({T}^{\ast }-T))}$. The resulting dimensionless free energy density (*f* → *fR*^2^/*k*_*i*_) reads

2 $$f=\frac{1}{2}\frac{\kappa }{{\kappa }_{i}}{g}_{H}+{\lambda }_{0}^{2}({(\frac{R}{\xi })}^{2}{g}_{c}+\frac{1}{2}{g}_{int}+\mu \,{g}_{ext}).$$

Here $${g}_{H}=Tr{\underline{C}}^{2}$$, $$\,{g}_{c}=-\,Tr{\underline{Q}}^{2}+\frac{1}{4}{(Tr{\underline{Q}}^{2})}^{2}$$, $${g}_{int}={|{\nabla }_{s}\underline{Q}|}^{2}$$, $${g}_{ext}=\underline{Q}\cdot {\underline{C}}^{2}$$, and $$\mu =\frac{{k}_{e}}{{\lambda }_{0}{k}_{i}}$$.

Note that in this scaling, the extrinsic term is weighted against the intrinsic term by a dimensionless coefficient $\mu \propto {\lambda }_{0}^{-1}$. Therefore, its contributions tend to diverge relative to the intrinsic term on approaching *T*_*c*_ from below.

### Curvature potentials

The relative importance of intrinsic and extrinsic elastic energies can be inferred from the positions of TDs. To this end, below we introduce geometric curvature potentials which highlight on the vesicle both attracting and repelling regions for TDs. These potentials are calculated for a given local curvature of the vesicle without solving the Euler-Lagrange equations for the equilibrium orientational ordering.

In order to define curvature potentials we first need to identify local ground states in 2D systems. In flat geometry in equilibrium, all elastic penalties vanish, i.e. ${g}_{\mathrm{int}}={g}_{ext}=0$. However, in curved manifolds the local ground state might carry a finite elastic penalty, to which we henceforth refer as the *fossil elastic energy*. Namely, it follows from the condition $\overrightarrow{n}\cdot \overrightarrow{v}=0$ that ${({\nabla }_{s}\overrightarrow{n})}^{T}\overrightarrow{v}=-\,\underline{C}\overrightarrow{n}$ and consequently in general $\frac{1}{2}{g}_{int}+\mu {g}_{ext}\ne 0$.

To determine local undistorted state of $\overrightarrow{n}$, we request that the director is parallel transported^56,58^ in all directions. If a unit vector ${\overrightarrow{e}}^{(p)}$ is locally parallel transported, it obeys the equation

3 $${\nabla }_{s}{\overrightarrow{e}}^{(p)}=-\,\overrightarrow{v}\otimes \underline{C}{\overrightarrow{e}}^{(p)},$$

where the superscript (p) indicates that a vector is parallel transported. Accordingly, we introduce the parallel transported nematic order tensor by ${\underline{Q}}^{(p)}={\overrightarrow{n}}^{(p)}\otimes {\overrightarrow{n}}^{(p)}-{\overrightarrow{n}}_{\perp }^{(p)}\otimes {\overrightarrow{n}}_{\perp }^{(p)}$, where a constant amplitude *λ*_0_ is understood. It follows that

4a $${w}_{int}\,:={g}_{int}^{(p)}={|{\nabla }_{s}{\underline{Q}}^{(p)}|}^{2}={C}_{1}^{2}+{C}_{2}^{2},$$

4b $${w}_{ext}\,:={g}_{ext}^{(p)}={\underline{Q}}^{(p)}\cdot {\underline{C}}^{2}=({C}_{1}^{2}-{C}_{2}^{2})\cos (2\vartheta ),$$

where $\overrightarrow{n}={\overrightarrow{e}}_{1}\,\cos \,\vartheta +{\overrightarrow{e}}_{2}\,\sin \,\vartheta$ is expressed in the eigenframe of $\underline{C}.$ We refer to *w*_*int*_ and *w*_*ext*_ as the *intrinsic curvature potential* and the *extrinsic curvature potential*, respectively. Note that *w*_*int*_ is independent of $\vartheta$. For a positive value of *k*_*e*_ (*μ* > 0), the *extrinsic curvature potential* tends to align $\overrightarrow{n}$ along the principal direction exhibiting minimal absolute value of principal curvature, for which ${w}_{ext}^{(min)}=-\,|{C}_{1}^{2}-{C}_{2}^{2}|$. Furthermore, we define the total curvature potential as

5 $${w}_{t}=\frac{1}{2}{w}_{int}+\mu \,{w}_{ext}^{(min)},$$

where we set *λ*_0_ = 1/2. Note that a finite value of *w*_*t*_ renormalizes the phase transition temperature. For example, for *k*_*e*_ = 0 the total orientational ordering free energy density due to a parallel transported nematic structure reads as $f={\alpha }_{0}(T-{T}_{eff}^{\ast })$ $Tr{\underline{Q}}^{2}+\frac{\beta }{4}$ ${(Tr{\underline{Q}}^{2})}^{2}$, where ${T}_{eff}^{\ast }={T}^{\ast }-\frac{{k}_{i}}{4{\alpha }_{0}}{w}_{int}$.

We now illustrate the prediction power of the potentials, thus defined. First, TDs tend to be *expelled* from regions where |*w*_*ext*_| is large enough (spatial variations of *w*_*ext*_ are presented in Supplementary Material). That is, the extrinsic term locally favors preferentially oriented structure, which is incompatible with spatially nonhomogeneous TD profiles. Second, in regions where *w*_*ext*_ is nearly uniform (or experiences relatively weak spatial variations), places exhibiting positive maxima in *w*_*t*_ tend to attract TDs in order to reduce condensation penalties enforced by TDs cores.

### Simulation results and discussion

Configurations of nematic vesicles are calculated numerically by minimizing the free energy for fixed values of the vesicle area and volume. Our study is limited to the larger values of reduced volume *v* where the discocyte RBCs were experimentally observed.

**Figure 3:**
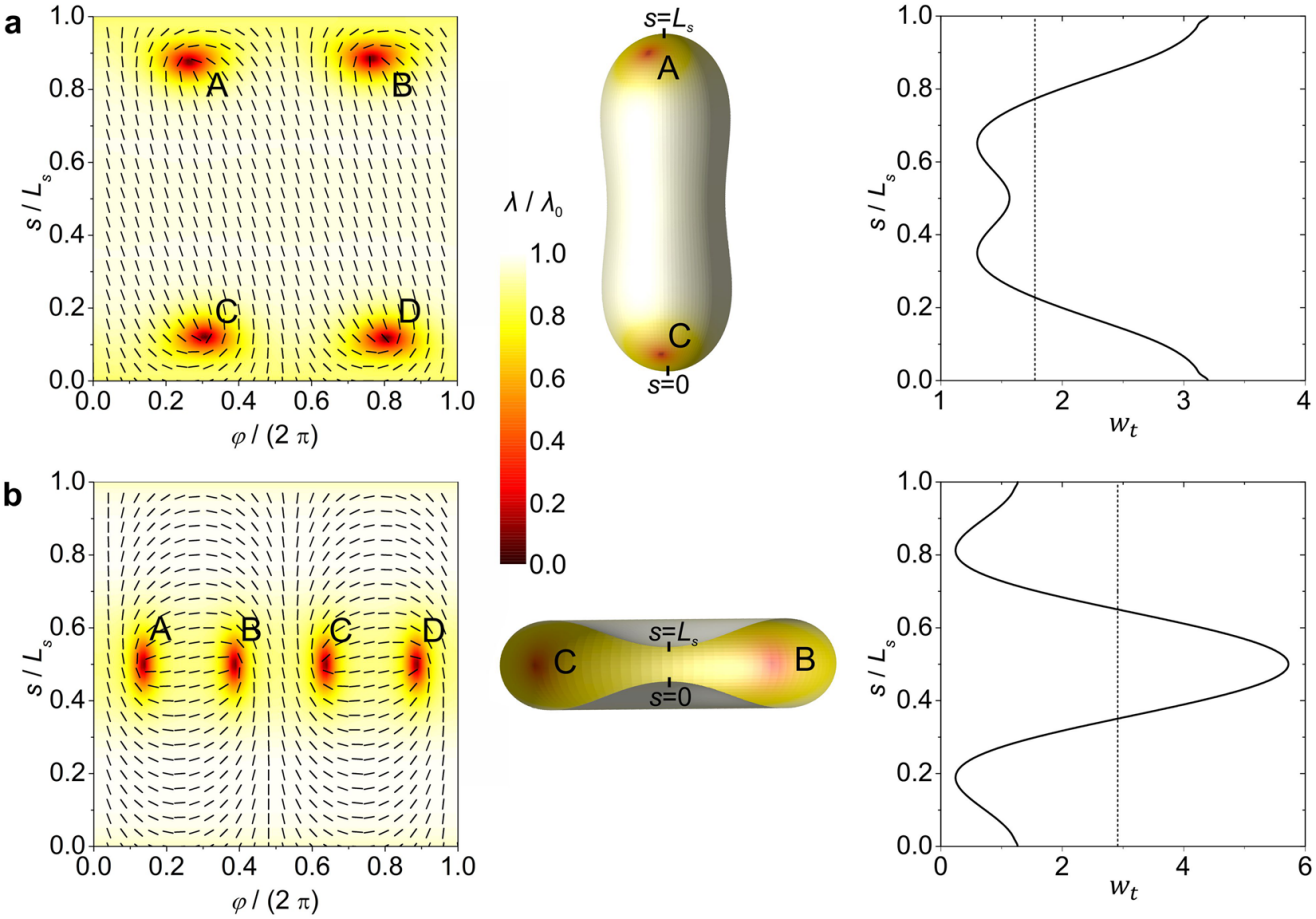
*Intrinsic* curvature dominated order parameter profiles with the corresponding *w*_*t*_(*s*) profile and equilibrium closed membrane shapes. Superimposed nematic director fields and order parameter profiles *λ* in the (*φ*, *s*)-plane are presented on the left, shell’s shapes with the corresponding order parameter profiles are presented in the middle, and *w*_*t*_ as a function of the arc length *s* (for *λ*_0_ = 1/2) is presented on the right. *L*_*s*_ stands for the length of the profile curve. Positions of TDs are denoted with capital letters. (**a)** *v* = 0.80, (**b**) *v* = 0.60, *R*/*ξ* = 7, *k*_*e*_ = 0, *k*_i_ = к.

Solutions found in our model therefore comprise either prolate or discocyte vesicle shapes, which are shown in the middle vertical panel of Fig. 3. Of particular interest for us is the stability regime of the discocyte shapes as a function of the reduced volume *v* and the ratio $\mu =\frac{{k}_{e}}{{\lambda }_{0}{k}_{i}}$, measuring the relative weight of the extrinsic and intrinsic elastic contributions.

**Figure 4:**
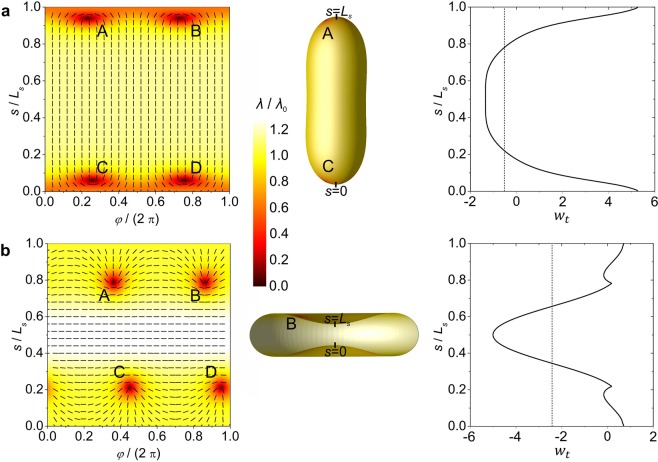
Impact of the *extrinsic* curvature term on order parameter profiles, equilibrium closed membrane shapes and *w*_*t*_(*s*). Left: superimposed nematic director fields and order parameter profiles in the (*φ*, *s*)-plane. Middle: shapes of shells with superimposed order parameter profiles. Right: *w*_*t*_(*s*) dependence for *λ*_0_ = 1/2. *L*_*s*_ stands for the length of the profile curve. Topological defects are denoted with capital letters. (**a**) *v* = 0.80, (**b**) *v* = 0.60. *R*/*ξ* = 7, *k*_e_ = *k*_i_/2, *k*_i_ = к.

For a reference, we first consider the case *k*_*e*_ = 0 (*μ* = 0), where the extrinsic (deviatoric) elasticity is absent. The representative structures and their characteristic features are given in Fig. 3. The corresponding structures calculated for *μ* = 1 are plotted in Fig. 4. In the first panel, we plot the order parameter as a function of the meridian arc length *s* and the azimuthal angle *φ*. We superimpose the order parameter amplitude and the director field spatial arrangement. Note that in our model we restrict to axially symmetric shapes, while in calculating the order tensor profile we allow for a fully 2D spatial dependence, i.e., $\underline{Q}=\underline{Q}(s,\varphi )$. In the second panel of Figs. 3 and 4, we plot vesicle shapes and in the third panel the total curvature potential. In Fig. 3a (top horizontal panel), we consider a prolate structure. For the studied set of parameters, it possesses four *m* = 1/2 TDs. These are assembled in regions exhibiting maximal positive Gaussian curvature in line with the ETCC mechanism. Positions of TDs are well visible in the first panel of the figure due to the melted core of defects. In Fig. 3b, we show a typical order parameter pattern, vesicle shape and *w*_*t*_(*s*) for discocytes. In these type of structures, four TDs are assembled along the equatorial line in order to screen the negative Gaussian curvature charge, which is localized in the equatorial region. Note that the *w*_*t*_(*s*) plot also predicts well the positions of TDs, which tend to be assembled in the areas where *w*_*t*_(*s*) exhibits its maximum value. Precisely, in oblate structures TDs are localized exactly at the *w*_*t*_(*s*) maximum. In prolate structures TDs are slightly below the poles, where *w*_*t*_(*s*) reaches its maximum, due to the mutual repulsion of TDs bearing the same topological charge.

In Fig. 4, we show the characteristic behavior of competing vesicle configurations for *μ* = 1, for which the relative weights of intrinsic and extrinsic (deviatoric) curvature terms are comparable. One sees (Fig. 4a) that prolate configurations are relatively weakly affected by the extrinsic contribution. The extrinsic (deviatoric) curvature potential (see Supplementary Material) is present everywhere except at the poles. Consequently, it pushes TDs towards the poles. The impact of the extrinsic curvature is relatively weak because in prolate structures, both intrinsic and extrinsic terms enforce similar attracting areas for TDs.

On the contrary, the extrinsic (deviatoric) curvature can substantially affect defect structures in discocytes. Precisely, the extrinsic curvature potential is finite in the equatorial region (see Supplementary Material), where defects tends to reside for *k*_*e*_ = 0 as predicted by the ETCC mechanism. However, strong enough extrinsic contribution expels TDs from this region, which is well visible in the 1^st^ plot of Fig. 4b. Furthermore, note that the profile of *w*_*t*_(*s*) (Eq. (5)), plotted in the Figs. 3 and 4, predicts well the locations of TDs. The graph reveals that TDs assemble at the local maximum in *w*_*t*_(*s*). Note that TDs do not assemble at the global maximum of *w*_*t*_(*s*) due to the mutual repulsion of TDs bearing the same topological charge.

**Figure 5:**
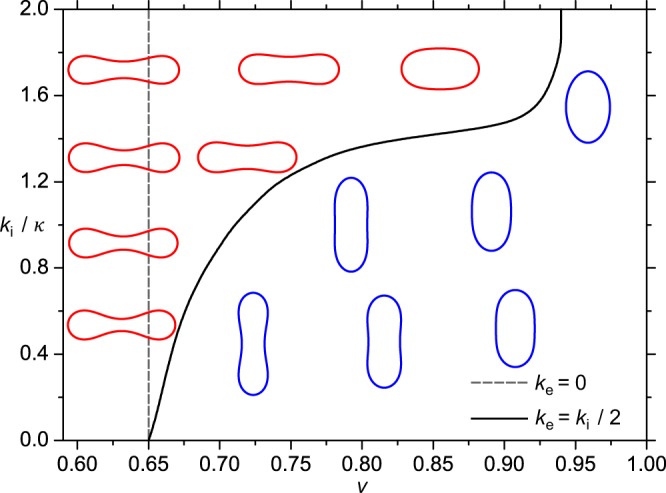
Equilibrium phase diagram of *nematic vesicles* in either the presence or absence of the *extrinsic* curvature term. The solid and dashed lines separate the stability regimes of the competing structures for *k*_e_ = *k*_i_/2 and *k*_e_ = 0, respectively. Oblate shapes are stable on the left side and prolate shapes on the right side of these lines. Stable shapes presented in the diagram are calculated in the presence of the *extrinsic* term (*k*_e_ = *k*_i_/2). They are shown for different values of the reduced volume *v* and *k*_i_/к. Prolate shapes are coloured in blue, oblate shapes (including discocyte shapes) are coloured in red *R*/*ξ* = 7.

The stability range as a function of *v* and elastic properties of nematic vesicles is shown in Fig. 5. One sees that discocytes are stable within a relatively narrow window of *v* values if the extrinsic (deviatoric) elasticity is neglected. For all ratios *k*_*i*_/*κ* studied, these structures are stable up to *v*_2_ ~ 0.65. When the extrinsic contribution is switched on, it significantly widens the stability window of discocyte and oblate shapes. For example, for $\frac{{k}_{i}}{\kappa }=1.4$ and $\frac{{k}_{e}}{{k}_{i}}=0.5$, we obtain *v*_2_ ~ 0.83, thus recovering the experimentally observed regime.

Therefore, our simulations reveal that the stability window of discocytes is significantly increased if the elastic constants entering our model are comparable. According to Fig. 5, the effects are significant for *κ* ~ *k*_*i*_ and *k*_*e*_ ≥ *k*_*i*_/2. Note that according to rough estimates (see Eq. (S5) in Supplementary Material and ref. ^42^) one expects *k*_*i*_ ~ *k*_*e*_, which justifies the predicted orientational order-driven broadening if *κ* ~ *k*_*i*_. In addition, on symmetry grounds it follows that also for cases ${k}_{i}\gg {k}_{e}$ the extrinsic elasticity could play an important role close to the orientational order-disorder phase transition. However, in this case the effect should be strongly temperature dependent.

Broader stability range of discocytes in the presence of extrinsic curvature is a consequence of their unique shape. The equatorial region of discocytes possesses a large difference between the principal curvatures *C*_1_ and *C*_2_. In the presence of extrinsic term, such surface patches are energetically favorable because they enforce strong orientational order, which contributes to the lower total free energy. This is clearly visible in the left panel of Fig. 4b, where strong orientational ordering is present in the equatorial region of the discocyte (see color plot). This region possesses stronger order than any region on a prolate shape shown in Fig. 4a. For this reason, discocyte shapes become energetically more favorable than prolate shapes in a wider window of *v* values when the impact of extrinsic curvature is taken into account, which is clearly visible in Fig. 5. Furthermore, our simulations reveal that the extrinsic curvature makes the free energy costs of the competing prolate and oblate structures comparable (see Fig. S3 in Supplementary Material). Therefore, this geometrically based phenomenon enables efficient switching between the structures. Note that this switching capability is evolutionary favorable because it enables RGBs to readily accommodate their shape in response to eventual temporally imposed stresses (e.g. in RGBs transport through capillaries).

The theoretical results presented in Fig. 5 may be influenced by consideration of thermal fluctuations of the RBC membrane, which were not taken into account in our approach. The thermal shape fluctuations of RBCs were first considered by Brochard and Lennon^59^. Later, the theory of membrane fluctuations applied on nearly spherical giant lipid vesicles was further developed by Helfrich^60^ and Milner and Safran^61^ and applied by many other authors^62–65^, which all used the mean field approximation. Using Monte Carlo simulations, it was recently shown^66^ that the errors made due the mean field approximations applied in the theory of membrane fluctuations are not very large. Comparison of the results of Monte Carlo simulations and the numerical minimization of the isotropic bending energy of closed membrane structures also indicated that thermal fluctuations do not significantly change the calculated closed membrane shapes^67^. However, in the case of orientational (nematic) ordering of membrane components, considered in the present paper, it is expected that the influence of membrane fluctuations on the calculated RBC shapes is slightly stronger, but still not an essential factor in determining the RBC shape. It can be also expected that nematic order would affect the non-spherical fluctuation modes of RBC membrane.

Expected values of the nematic elastic constants, which would according to our simulations enhance the stability window of discocyte RBC shapes, are as follows. Typical value of the bending modulus of RBCs is $\kappa  \sim 1.8\cdot {10}^{-19}$ J^68^. Furthermore, in experiments with giant unilamellar vesicles, which are often used to study biological systems and to mimic cell membranes, the bending modulus of membranes ranges from $\kappa  \sim 0.25\cdot {10}^{-19}$ J to $\kappa  \sim 2.1\cdot {10}^{-19}$ J^64^. Our simulations reveal that the extrinsic elastic constant *k*_*e*_ should be of the same order of magnitude as *κ* in order to substantially increase the *v* window of stability of discocytes. Therefore, *k*_*e*_ > 10^−19^ J, which is much larger than the thermal fluctuations (e.g., at room temperature, ${k}_{B}T \sim 0.04\cdot {10}^{-19}$ J, where *k*_*B*_ is the Boltzmann constant).

Finally, it might be that RBCs exhibit hexagonal bond order. In this case, the winding number of “elementary” topological defects equals *m* = ±1/6. Our approach is qualitatively valid also for such ordering due to the topological origin of the phenomenon. However, the free energy expression would be different because of different order parameter describing the ordering. Consequently, the stability diagram could be quantitatively different.

## Conclusions

In conclusion, we consider theoretically the stability of RBC configurations focusing on the competition between prolate and oblate-like shapes. Existent mesoscopic approaches fail to explain a relatively broad range of relative volumes $v\in [{v}_{1},{v}_{2}]$ for which oblate discocytes are experimentally observed. We demonstrate that taking into account the in-plane ordering and extrinsic (deviatoric) curvature elasticity, the value of *v*_2_ could be efficiently increased, stabilising discocytes within experimentally observed values of *v*. In particular, nematic-like ordering could be present due to flexible hydrocarbon chains of lipids or due to anisotropic proteins embedded within membranes. Note that the extrinsic curvature has been traditionally discarded in theoretical studies without a reasonable justification. On the other hand, a simple modelling reveals that the elastic moduli measuring the relative strength of intrinsic and extrinsic curvature contributions are at least comparable. We show that for the nematic-type of ordering, the ratio $\mu =\frac{{k}_{e}}{{\lambda }_{0}{k}_{i}}$ between coefficients measuring the extrinsic and intrinsic contributions is inversely proportional with the orientational order parameter. Consequently, the extrinsic curvature is expected to dominate orientational ordering on approaching the order-disorder phase transition temperature from below for a 2^nd^ order or weakly 1^st^ order phase transition. In particular, our model shows that the extrinsic (deviatoric) curvature strongly increases the stability regime of oblate RBC structures. Furthermore, in this regime the free energies of the competing oblate and prolate structures are almost comparable. It is reasonable that the natural evolution tuned membrane parameters to such a regime to enable sensitive structural responsivity of RBC shapes, enabling their efficient transport to various parts of biological tissues. Note that living creatures and their constituents often self organize at the edge of a phase transition of a relevant emerging macroscopic order. For example, the mechanical theory of information propagation in nerves^69^ assumes that the nerve membrane is just above the liquid-liquid crystal phase transition of membrane lipid constituents. Such tuning to the edge of phase transition equips systems with sensitive response to external stimuli.

Furthermore, the relative weight of extrinsic and intrinsic curvatures is transparently presented in curvature potentials which we introduce using the parallel transport method. They are determined solely by geometry. In particular, we show that taking into account both the extrinsic potential and total curvature potential one could predict regions where TDs assemble without solving the corresponding equilibrium equations determining structural ordering. Thus, this new approach upgrades the existent ETCC mechanism^50^ which takes into account only the intrinsic contribution.

## Methods

In simulations, we considered closed axisymmetric two-dimensional (2D) shells exhibiting spherical topology. Shell surface is assumed to be a surface of revolution with rotational symmetry about the z-axis within the Cartesian system (*x*, *y*, *z*), which is defined by the unit vectors (${\overrightarrow{e}}_{x}$, ${\overrightarrow{e}}_{y}$, ${\overrightarrow{e}}_{z}$). Such surfaces are constructed by the rotation of the profile curve about the ${\overrightarrow{e}}_{z}$ axis by an angle of *φ* = 2*π*. A generic point lying on an axisymmetric surface is given by^50^:

6 $$\overrightarrow{r}=\rho (s)\cos \,\varphi {\overrightarrow{e}}_{x}+\rho (s)\sin \,\varphi {\overrightarrow{e}}_{y}+z(s){\overrightarrow{e}}_{z},$$

where *ρ*(*s*) and *z*(*s*) are the coordinates of the profile in the (*ρ*, *z*)-plane and *s* represents the arc length of the profile curve. On a surface of revolution, parallels and meridians are lines of principal curvature. We define that the principal directions (${\overrightarrow{e}}_{1},\,{\overrightarrow{e}}_{2}$) (see Eq. (1a)) point along meridians (*φ* = *const*) and parallels (*s* = *const*), respectively.

### Calculation of profile curve

In order to calculate shell shapes within our hybrid Helfrich-Landau mesoscopic approach, we introduce an angle *θ*(*s*), which represents the angle of the tangent to the profile curve with the plane that is perpendicular to the axis of rotation ${\overrightarrow{e}}_{z}$. The profile curve of axisymmetric surface is calculated by^50,70–72^:

7 $$\rho (s)={\int }_{0}^{s}\,\cos \,\theta (s^{\prime} ){\rm{d}}s^{\prime} ,\,z(s)={\int }_{0}^{s}\,\sin \,\theta (s^{\prime} ){\rm{d}}s^{\prime} .$$

The boundary conditions for closed and smooth surfaces are as follows: *θ*(0) = 0, *θ*(*L*_*s*_) = *π*, $\rho (0)=\rho ({L}_{s})=0$, where *L*_*s*_ stands for the length of the profile curve^50,70–72^. Furthermore, the function describing the angle *θ*(*s*) is approximated by the Fourier series^50,70–72^:

8 $$\theta (s)={\theta }_{0}\frac{s}{{L}_{s}}+\mathop{\sum }\limits_{i=1}^{N}\,{a}_{i}\,\sin (\frac{\pi }{{L}_{s}}i\cdot s),$$

where *N* represents the number of Fourier modes, *a*_*i*_ are the Fourier amplitudes, and ${\theta }_{0}=\theta ({L}_{s})=\pi$ is the angle at the north pole of the axisymmetric surface. The local principal curvatures *C*_1_ and *C*_2_ are given as $\frac{d\theta (s)}{ds}$ and $\frac{\sin \,\theta (s)}{\rho (s)}$, respectively^50^.

### Calculation of nematic order

Nematic order tensor (Eq. (1b)) is parameterized as^50,55,56^:

9 $$\underline{Q}={q}_{1}({\overrightarrow{e}}_{1}\otimes {\overrightarrow{e}}_{1}-{\overrightarrow{e}}_{2}\otimes {\overrightarrow{e}}_{2})+{q}_{2}({\overrightarrow{e}}_{1}\otimes {\overrightarrow{e}}_{2}+{\overrightarrow{e}}_{2}\otimes {\overrightarrow{e}}_{1}),$$

where *q*_1_ and *q*_2_ are scalar functions. The standard functions that represent the first fundamental form on axisymmetric surfaces in the $(\varphi ,s)$ coordinates are given as^50,55,73^:

10 $$E\,:={\overrightarrow{r}}_{,\varphi }\cdot {\overrightarrow{r}}_{,\varphi }=\rho {(s)}^{2},\,F\cdot {\overrightarrow{r}}_{,\varphi }\cdot {\overrightarrow{r}}_{,s}=0,\,G\cdot {\overrightarrow{r}}_{,s}\cdot {\overrightarrow{r}}_{,s}=\rho {(s)}_{,s}^{2}+z{(s)}_{,s}^{2},$$

where vector $\overrightarrow{r}$ is defined by Eq. (6) and a comma denotes the differentiation. Furthermore, Jacobian determinant is given by:

11 $$J(s)\,:\,=\sqrt{EG-{F}^{2}}=\rho (s)\sqrt{\rho {(s)}_{,s}^{2}+z{(s)}_{,s}^{2}}.$$

On a surface of revolution, the meridians are also geodesics, so their geodesic curvature ${\kappa }_{g1}=0$, while the geodesic curvature of the parallels can be written as^50,55,73^:

12 $${\kappa }_{g2}=\frac{{E}_{,s}}{2E\sqrt{G}}=\frac{\rho {(s)}_{,s}}{\rho (s)\sqrt{\rho {(s)}_{,s}^{2}+z{(s)}_{,s}^{2}}}.$$

The Gaussian and the mean curvature of an axisymmetric surface are given as^50,55,73^:

13 $$K(s)=-\,\frac{z{(s)}_{,s}(z{(s)}_{,s}\rho {(s)}_{,ss}-z{(s)}_{,ss}\rho {(s)}_{,s})}{\rho (s){(\rho {(s)}_{,s}^{2}+z{(s)}_{,s}^{2})}^{2}},$$

14 $$H(s)=\frac{\rho (s)(z{(s)}_{,s}\rho {(s)}_{,ss}-z{(s)}_{,ss}\rho {(s)}_{,s})-z{(s)}_{,s}(\rho {(s)}_{,s}^{2}+z{(s)}_{,s}^{2})}{\rho (s){(\rho {(s)}_{,s}^{2}+z{(s)}_{,s}^{2})}^{3/2}}.$$

Note that *K* and *H* are connected to the local principal curvatures via

15 $$K={C}_{1}{C}_{2},\,2H={C}_{1}+{C}_{2},$$

The surface gradient of a scalar function in the coordinates (*φ*, *s*) on an axisymmetric surface is given by^50^:

16 $${\nabla }_{s}\varphi =\frac{1}{\sqrt{G}}\frac{\partial \varphi }{\partial s}{\overrightarrow{e}}_{1}+\frac{1}{\rho (s)}\frac{\partial \varphi }{\partial \varphi }{\overrightarrow{e}}_{2}$$

while the surface gradients of ${\overrightarrow{e}}_{1}$ and ${\overrightarrow{e}}_{2}$ are given by Eqs. (S4a) and (S4b) (see Supplementary Material). For a given closed surface geometry, the free energy density associated with nematic ordering is expressed in terms of fields *q*_1_ and *q*_2_.

### Numerical simulations

In simulations, we determine equilibrium closed shell shapes and nematic ordering textures on these shells. Equilibrium nematic textures are calculated by using the standard Monte Carlo method, while equilibrium shell shapes are calculated by the numerical minimisation of the function of many variables^70–72^. Equilibrium nematic configuration is calculated with Monte Carlo method on a fixed shape. In the next step, equilibrium surface shape is adjusted according to the current nematic texture. This process is repeated until the equilibrium shell shape and equilibrium nematic texture are obtained.

The bending energy density *f*_*H*_ of the deformable surface is a function of the Fourier amplitudes *a*_*i*_ and the shape profile length *L*_*s*_ (see Eq. (8)). Equilibrium shell shapes were calculated by the numerical minimisation of the function of many variables^70–72^. During the minimisation procedure, the shell surface area *A* and the volume *V* were kept constant in order to set a fixed value of the shell reduced volume *v*.

Equilibrium nematic textures were calculated with Monte Carlo method. The shell surface in the coordinates (*φ*, *s*) is represented as the network of 101 × 101 points. At each point, the free energy density *f* = *f*_*c*_ + *f*_*e*_ associated with nematic ordering is calculated numerically. With the aid of the Jacobian determinant *J*(*s*) (Eq. (11)), the numerical integration is performed over the shell surface in order to obtain the total free energy associated with nematic ordering.

## Supplementary information

Supplementary material
