# Supplementary material for: Normal red blood cells’ shape stabilized by membrane’s in-plane ordering
Source: Sci Rep. 2019 Dec 24;9:19742. doi: 10.1038/s41598-019-56128-0 (PMC6930264; doi:10.1038/s41598-019-56128-0)
Supplement: Supplementary file 1 — Supplementary material [file 41598_2019_56128_MOESM1_ESM.docx]

**Normal red blood cells' shape stabilized by membrane's in-plane ordering**

L. Mesarec^1^, W. Góźdź^2^, A. Iglič^1,3^, V. Kralj-Iglič^3,4,5^, E. G. Virga^6^ and S. Kralj^7,8,*^

*^1^ Laboratory of Biophysics, Faculty of Electrical Engineering, University of Ljubljana, 1000 Ljubljana, Slovenia*

*^2^ Institute of Physical Chemistry, Polish Academy of Sciences, 01‑224 Warsaw, Poland*

*^3^ Laboratory of Mass Spectrometry and Proteomics, Institute of Biosciences and BioResources, National Research Council of Italy, Napoli 80132, Italy*

*^4^ Laboratory of Clinical Biophysics, Faculty of Health Sciences, University of Ljubljana, 1000 Ljubljana, Slovenia*

*^5^ Laboratory of Clinical Biophysics, Faculty of Medicine, University of Ljubljana, 1000 Ljubljana, Slovenia*

*^6^ Department of Mathematics, University of Pavia, Via Ferrata 5, 27100 Pavia, Italy*

*^7^ Department of Physics, Faculty of Natural Sciences and Mathematics, University of Maribor, 2000 Maribor, Slovenia*

*^8^ Condensed Matter Physics Department, Jožef Stefan Institute, 1000 Ljubljana, Slovenia*

*^*^ Corresponding author: samo.kralj@um.si*

**Supplementary material**

1. *Intrinsic* and *extrinsic* curvature

In the following, by using a minimalist model, we show that the elastic moduli weighting the strength of intrinsic and extrinsic curvature contributions are expected to be comparable.

To this purpose, we consider a 2D nematic film on a curved substrate. In the simplest possible model, we penalize elastic distortions with the elastic free energy density term

$f=k\left| \nabla_{s}\vec{n} \right|^{2}$, (S1)

where $k$ is a positive elastic constant. We parametrize $\vec{n}$ in the substrate principal curvature frame ($\vec{e}_{1},\vec{e}_{2}$):

$\vec{n}=\vec{e}_{1}cos\vartheta+\vec{e}_{2}sin\vartheta$. (S2)

It follows that

$\nabla_{s}\vec{n}= cos\vartheta\nabla_{s}\vec{e}_{1}-sin\vartheta{\vec{e}_{1}\otimes\nabla}_{s}\vartheta+sin\vartheta\nabla_{s}\vec{e}_{2}+cos\vartheta{\vec{e}_{2}\otimes\nabla}_{s}\vartheta$. (S3)

Taking into account that [1]

$\nabla_{s}\vec{e}_{1}=\kappa_{g1}\vec{e}_{2}\otimes\vec{e}_{1}{+\kappa_{g2}\vec{e}}_{2}\otimes\vec{e}_{2}-C_{1}\vec{v}\otimes\vec{e}_{1}$, (S4a)

$\nabla_{s}\vec{e}_{2}=-\kappa_{g1}\vec{e}_{1}\otimes\vec{e}_{1}{-\kappa_{g2}\vec{e}}_{1}\otimes\vec{e}_{2}-C_{2}\vec{v}\otimes\vec{e}_{2}$, (S4b)

where $\kappa_{g1}$ and $\kappa_{g2}$ are the geodesic curvatures along $\vec{e}_{1}$ and $\vec{e}_{2}$, we obtain

$\left| \nabla_{s}\vec{n} \right|^{2}=\left| \nabla_{s}\vartheta+\vec{A} \right|^{2}+\vec{n}\cdot\underline{C}^{2}\vec{n}$. (S5)

The quantity $\vec{A}=\kappa_{g1}\vec{e}_{1}+\kappa_{g2}\vec{e}_{2}$ is referred to as the spin connection [2,3] and it satisfies $K=\nabla_{s}\times\vec{A}\cdot\vec{v}$, where $\vec{v}=\vec{e}_{1}\times\vec{e}_{2}$ is the outer unit normal to the nematic vesicle.

One can express $f$ as the sum $f=f^{(int)}+f^{(ext)}$, where

$f^{(int)}=k\left| \nabla_{s}\vartheta+\vec{A} \right|^{2}$, (S6a)

$f^{(ext)}=k\vec{n}. \underline{C}^{2}\vec{n}=k\left( C_{1}^{2}{cos}^{2}\vartheta+C_{2}^{2}{sin}^{2}\vartheta\right)$. (S6b)

The *intrinsic* term ($f^{(int)}$) is minimized if $\vec{n}$ is locally parallel transported [1], i.e. if $\nabla_{s}\vec{n}=-(\vec{v}\otimes\underline{C}$)$\vec{n}$. That is, using the parametrization in Eq. (S2), this condition yields $\nabla_{s}\vartheta=-\vec{A}{=-(\kappa}_{g1}\vec{e}_{1}+\kappa_{g2}\vec{e}_{2})$, and consequently $f^{(int)}=0$, which again is meant to be valid locally (not necessarily on the whole surface). One also sees that the *extrinsic* term ($f^{(ext)}$) acts as an effective external field, tending to align $\vec{n}$ along the principal direction exhibiting minimal absolute curvature.

In this model, both terms are weighted by the same elastic constant $k$.

1. **Curvature potentials**

In this section, we derive the appropriate form of curvature potentials in our Landau-type approach. Symmetry allowed elastic free energy density contributions expressed in terms of $\underline{Q}$ read as

$f_{e}=L_{1}\left| \nabla_{s}\underline{Q} \right|^{2}+L_{2}\left( \nabla_{s}\cdot\underline{Q} \right)^{2}+L_{3}\nabla_{s}\underline{Q}\cdot{\nabla_{s}\underline{Q}}^{T}$, (S7)

where $L_{1}$, $L_{2}$, $L_{3}$ are bare (temperature independent) nematic elastic constants [1,4]. Next, we express $f_{e}$ for a locally parallel transported nematic state. Imposing $\underline{Q}=\underline{Q}^{(p)}$, where $\underline{Q}^{(p)}$ is defined in the main text, it follows that

$$f_{e}^{\left( p \right)}=\frac{L_{1}}{2}{Tr\underline{Q}^{\left( p \right)}}^{2}Tr\underline{C}^{2}+L_{3}K{Tr\underline{Q}^{\left( p \right)}}^{2}+$$

$\left( L_{2}+L_{3} \right)\left( \underline{Q}^{\left( p \right)}\cdot\underline{C} \right)^{2}+\left( 2L_{1}{+L}_{2}+L_{3} \right)\underline{Q}^{\left( p \right)}\cdot\underline{C}^{2}$. (S8)

We introduce intrinsic ($w_{int}$) and extrinsic ($w_{ext}$) curvature potentials via

$f_{e}^{(p)}=\frac{k_{i}}{2}w_{int}+k_{e}w_{ext}$, (S9)

where

$w_{int}=\frac{1}{2}{Tr\underline{Q}^{\left( p \right)}}^{2}Tr\underline{C}^{2}{+L}_{3}/L_{1} K{Tr\underline{Q}^{\left( p \right)}}^{2}$, (S10a)

$w_{ext}=\underline{Q}^{\left( p \right)}\cdot\underline{C}^{2}+\left( L_{2}+L_{3} \right)/(2L_{1}{+L}_{2}+L_{3})\left( \underline{Q}^{\left( p \right)}\cdot\underline{C} \right)^{2}$, (S10b)

$k_{i}/2=L_{1}$, $k_{e}=2L_{1}{+L}_{2}+L_{3}$. (S10c)

In the main text, we only considered the essential elastic terms. To this purpose, here too we take into account only the terms that survive the approximation to a single elastic constant, that is, to setting $L_{2}=L_{3}=0$ and $L_{1}>0$ [4]. However, to keep some degree of freedom, we allow $k_{i}$ and $k_{e}$ to be independent. With these choices, Eqs. (S10a) and (S10b) are found to coincide with Eqs. (4a) and (4b) in the main text, after having observed that in our normalization ${Tr\underline{Q}}^{2}=2$.

1. **Analysis of curvature potentials**

In this section, we show how several qualitative features of a nematic vesicle could be predicted solely from the analysis of the behaviour of the intrinsic $w_{int}$ (Eq. (4a)) and extrinsic $w_{ext}$ (Eq. (4b)) curvature potentials. The extrinsic curvature potential $w_{ext}$ acts as a geometric field: it enforces preferred directions on the eigenframe of $\mathbf{Q}$. It favours alignment along the principal curvature direction exhibiting the lowest absolute curvature. On the other hand, the intrinsic curvature potential $w_{int}$ renormalizes the local temperature. If $w_{int}>0$ $(w_{int}<0)$ it locally prefers melting (increased degree of ordering). Therefore, positive maxima of $w_{int}$ act as attractive sites for topological defects (TDs). On the contrary, negative minima of $w_{int}$ repel defects. Note that $w_{ext}$ enforces orientation along a principal curvature (i.e. $\cos(2\vartheta)=\pm1$). In general, $w_{int}$ and $w_{ext}$ could induce antagonistic tendencies. In these cases, it is instructive to investigate the behaviour of $w_{t}$ (Eq. (5)), which gives information on their combined impact on the local degree of ordering for the case where the eigenframe of $\mathbf{Q}$ is aligned along the direction preferred by $w_{ext}$. Note that negative minima of $w_{t}$ correspond to an increased degree of ordering and therefore repel TDs. On the contrary, positive maxima of $w_{t}$ promote local melting and therefore attract TDs.

In Fig. S1, we plot the extrinsic curvature potential $w_{ext}$ (see Eq. (4b)) as a function of angle $\vartheta$ and arc length of the profile curve $s$ for prolate (Fig. S1a) and oblate (Fig. S1b) shape. At each value $s$, minima (maxima) of $w_{ext}$ identify energetically most favourable (unfavourable) nematic director orientation angles $\vartheta$. We observe that on a prolate shape (Fig. S1a), the orientation of director field along the meridians ($\vartheta=0$) is energetically most favourable on the majority of the surface, which was confirmed by our simulations in the presence of an *extrinsic* term (see Fig. 4a). On an oblate shape (Fig. S1b), the orientation of the director field along parallels ($\vartheta=\pi/2$) is energetically more favourable in the equatorial region (see also Fig. 4b), while the remaining part of the shape does not exhibit any distinct minima or maxima of $w_{ext}$. Due to such a strong *extrinsic* term enforcing orientational ordering, topological defects are expelled from the equatorial region of oblate shapes (Fig. 4b).

**
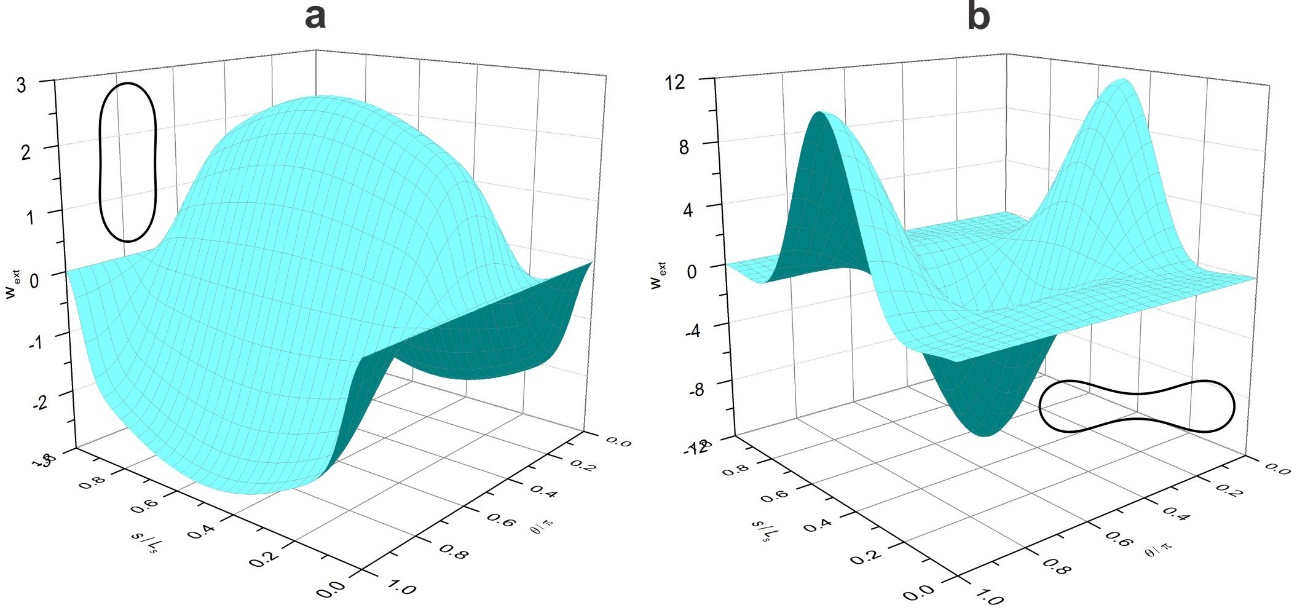
**

**Fig. S1** Extrinsic potential $w_{ext}$ for qualitatively different shapes. Plots of $w_{ext}$ as a function of the arc length $s$ and the nematic director orientation angle $\vartheta$ for **a)** prolate and **b)** oblate shapes. Calculations were performed for the shapes presented in Fig. 4. Shell profiles are given within graphs for each shape.

In Fig. S2, we study the impact of increasing the relative effect of the *extrinsic* term, i.e. increasing the $k_{e}/k_{i}$ ratio, on $w_{t}$ (see Eq. (5)) for prolate (Fig. S2a) and oblate (Fig. S2b) shape. On a prolate shape (Fig. S2a), there is no qualitative change in $w_{t}$ upon increasing the $k_{e}/k_{i}$ ratio. Two maxima of $w_{t}$ are located at the poles ($s=0$ and $s=L_{s}$), therefore, topological defects on a prolate shape are attracted to the poles (see Figs. 3a and 4a). On an oblate shape (Fig. S2b), the positions of maxima and minima of $w_{t}$ are altered upon increasing the $k_{e}/k_{i}$ ratio. In the absence of any *extrinsic* term ($k_{e}=0)$, the equator ($s=L_{s}/2$) corresponds to the maximum of $w_{t}$. Therefore, topological defects are located at the equator for $k_{e}=0$ (see Fig. 3b). However, upon increasing the $k_{e}/k_{i}$ ratio, the equator hosts the minimum of $w_{t}$, while two local maxima appear between the equator and each pole (Fig. S2b). Topological defects are in this case expelled from the equatorial region. They appear at the local maxima of $w_{t}$ between the equator and each pole (Fig. 4b). Note that even though global maxima of $w_{t}$ are located at the poles, topological defects are not located at the poles because of their mutual repulsion.


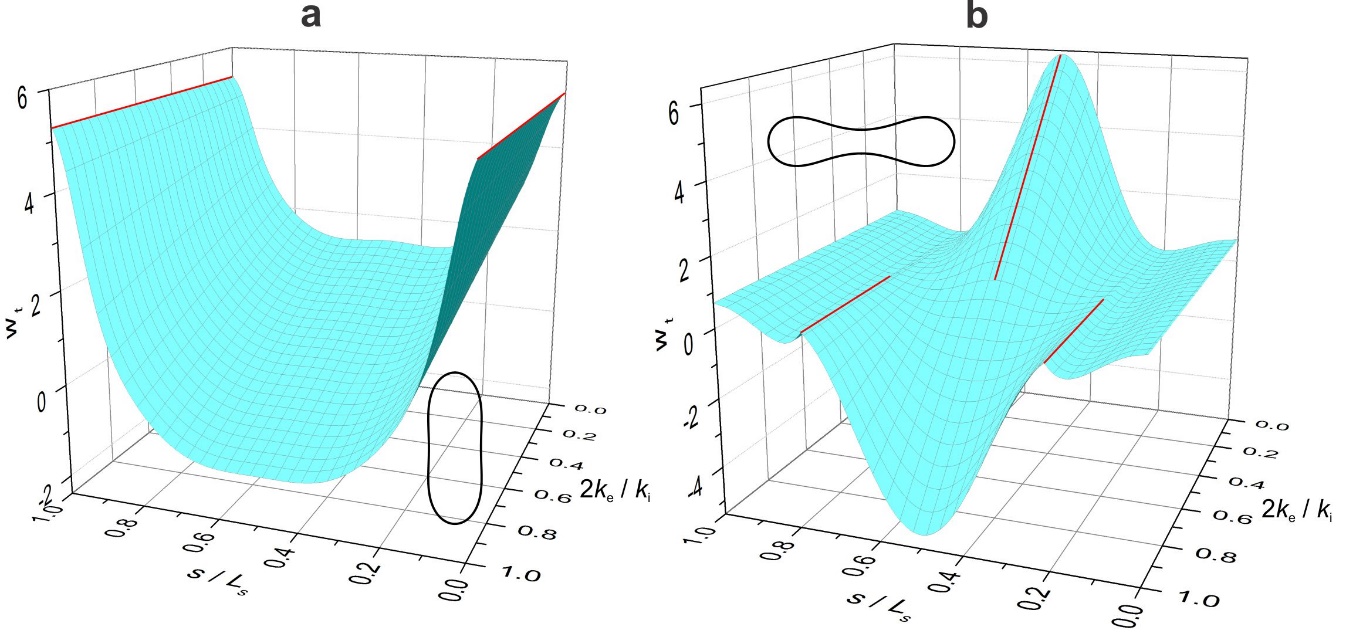


**Fig. S2** Behavior of $w_{t}$ upon increasing the relative weight of the *extrinsic* term. Plots of $w_{t}$ as a function of the arc length $s$ and the ratio ${2k}_{e}/k_{i}$ for **a)** prolate and **b)** oblate shapes. Calculations were performed for the shapes presented in Fig. 4. Global and local maxima of $w_{t}$ are denoted by red lines. Shell profiles for each shape are given within graphs.

1. **Total free energy**

The total free energy $F=\int fd^{2}\vec{r}$ as a function of the reduced volume $v$ is presented in Fig. S3. Here $f=f_{H}+f_{c}+f_{e}$ is defined as in the main part of the paper and the integral is computed over the whole closed membrane surface. Thin lines represent the case in the absence of the *extrinsic* term ($k_{e}=0)$, while bold lines are used to plot the energy values in the presence of the *extrinsic* term ($k_{e}=k_{i}/2$). In both cases, the coupling between the nematic in-plane orientational ordering and membrane curvature is set as $k_{i}/к=1.4$. At this coupling ratio, the border between oblate and prolate shapes is rapidly changing (see Fig. 5) upon varying $v$. For $k_{e}=0$ (thin lines in Fig. S3), the stability regime predicted by the classical Helfrich model is recovered [5,6] (see also Fig. 5). For $k_{e}=k_{i}/2$ (bold lines in Fig. S3) the stability region of oblate (discocyte) shapes is significantly increased. Note that the energies of oblate and prolate shapes are very similar for $0.75<v<1.0$. Therefore, in this regime the transformation between the competing shapes is easily realised.

**
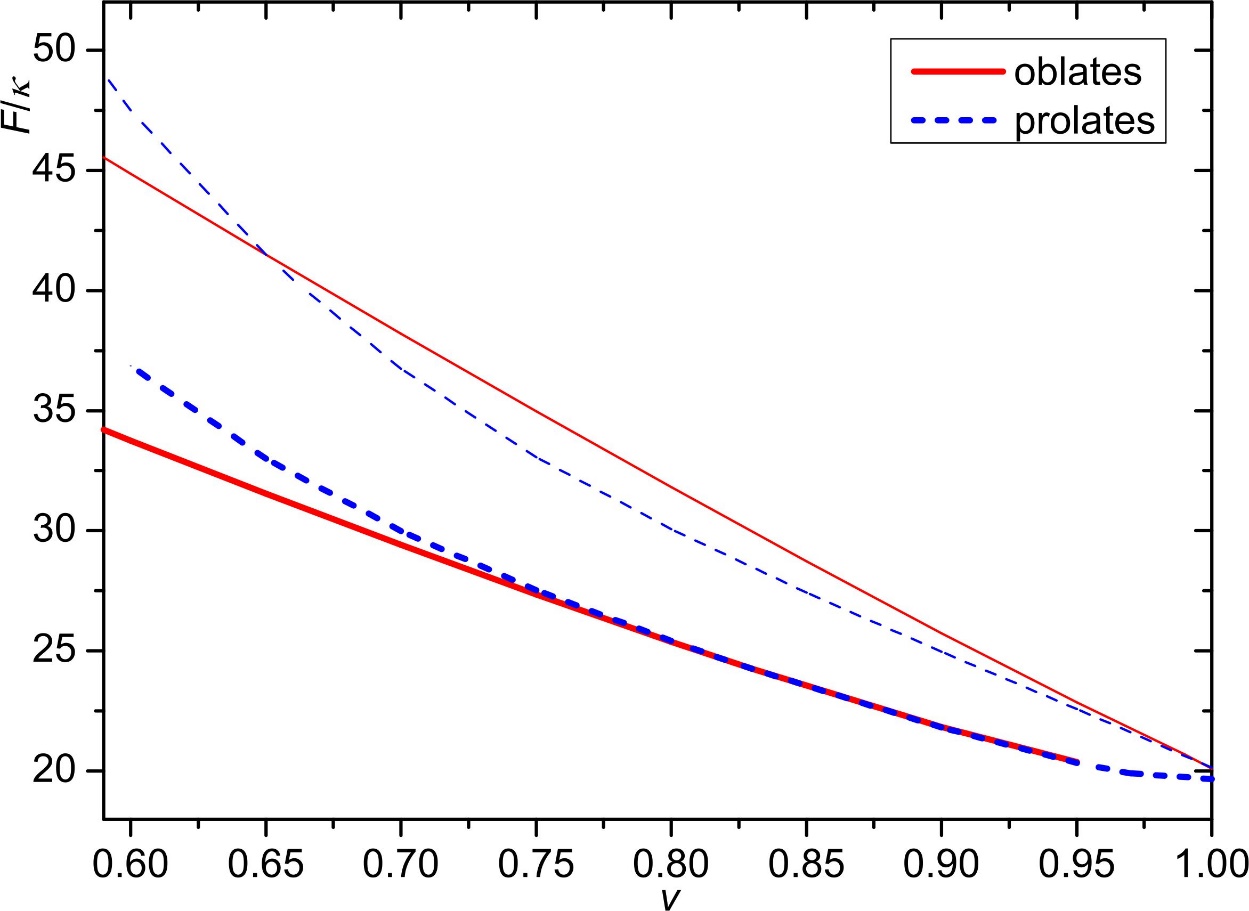
**

**Fig. S3** The total energy $F$ in units $к$ of closed membrane shapes as a function of reduced volume. The energy is calculated for $k_{e}=0$ (thin lines) and for $k_{e}=k_{i}/2$ (bold lines). Prolates are denoted with dashed lines and oblates with solid lines. $R/\xi=7$, $k_{i}/к=1.4$.

**References**

[1] Rosso, R., Virga, E. G. & Kralj, S. Parallel transport and defects on nematic shells. *Continuum Mech. Therm.* **24,** 643–664 (2012).

[2] Selinger, R. L. B., Konya, A., Travesset, A. & Selinger, J. V. Monte Carlo studies of the XY model on two-dimensional curved surfaces. *J. Phys. Chem. B* **115,** 13989-13993 (2011).

[3] Pairam, E. *et al.* Stable nematic droplets with handles. *Proc. Natl. Acad. Sci. USA* **110,** 9295-9300 (2013).

[4] Kralj, S., Rosso, R. & Virga, E. G. Curvature control of valence on nematic shells. *Soft Matter* **7,** 670–683 (2011).

[5] Seifert, U., Berndl, K. & Lipowsky, R. Shape transformations of vesicles: Phase diagram for spontaneous-curvature and bilayer-coupling models. *Phys. Rev. A* **44(2),** 1182 (1991).

[6] Bahrami, A. H. *et al.* Wrapping of nanoparticles by membranes. *Adv. Colloid Interface Sci.* **208,** 214-224 (2014).
